# Active smokers show ameliorated delayed gastric emptying after pancreatoduodenectomy

**DOI:** 10.1186/s12893-021-01311-2

**Published:** 2021-07-31

**Authors:** Jana Enderes, Jessica Teschke, Martin von Websky, Steffen Manekeller, Jörg C. Kalff, Tim R. Glowka

**Affiliations:** Department of Surgery, University Hospital Bonn, Venusberg-Campus 1, 53127 Bonn, Germany

**Keywords:** Pancreaticoduodenectomy, Whipple, Delayed gastric emptying, Nicotine, Smoking

## Abstract

**Background:**

Delayed gastric emptying (DGE) is the most common complication following pancreatoduodenectomy (PD). The data about active smoking in relation to gastric motility have been inconsistent and specifically the effect of smoking on gastric emptying after PD has not yet been investigated in detail.

**Methods:**

295 patients at our department underwent PD between January 2009 and December 2019. Patients were analyzed in relation to demographic factors, diagnosis, pre-existing conditions, intraoperative characteristics, hospital stay, mortality and postoperative complications with special emphasis on DGE. All complications were classified according to the definitions of the International Study Group on Pancreatic Surgery.

**Results:**

274 patients were included in the study and analyzed regarding their smoking habits (non or former smokers, n = 88, 32.1% vs. active smokers, n = 186, 68.6%). Excluded were patients for whom no information about their smoking habits was available (n = 3), patients who had had gastric resection before (n = 4) and patients with prolonged postoperative resumption to normal diet independently from DGE (long-term ventilation > 7 days, fasting due to pancreatic fistula) (n = 14). Smokers were younger than non-smokers (61 vs. 69 years, p ≤ 0.001) and mainly male (73% male vs. 27% female). Smoking patients showed significantly more pre-existing pulmonary conditions (19% vs. 8%, p = 0.002) and alcohol abuse (48% vs. 23%, p ≤ 0.001). We observe more blood loss in smokers (800 [500–1237.5] vs. 600 [400–1000], p = 0.039), however administration of erythrocyte concentrates did not differ between both groups (0 [0–2] vs. 0 [0–2], p = 0.501). 58 out of 88 smokers (66%) and 147 out of 186 of non-smokers (79%) showed malign tumors (p = 0.019). 35 out of 88 active smokers (40%) and 98 out of 188 non- or former smokers (53%) developed DGE after surgery (p = 0.046) and smokers tolerated solid food intake more quickly than non-smokers (postoperative day (POD7 vs. POD10, p = 0.004). Active smokers were less at risk to develop DGE (p = 0.051) whereas patients with pulmonary preexisting conditions were at higher risk for developing DGE (p = 0.011).

**Conclusions:**

Our data show that DGE occurs less common in active smokers and they tolerate solid food intake more quickly than non-smokers. Further observation studies and randomized, controlled multicentre studies without the deleterious effect of smoking, for instance by administration of a nicotine patch, are needed to examine if this effect is due to nicotine administration.

## Background

Pancreatic ductal adenocarcinoma (PDAC) is one of the most aggressive solid tumors with a very poor prognosis estimated to be the second most cause of cancer related-deaths in 2030 in both male and female [1]. Surgical resection is the only potentially curative therapy for PDAC followed by an adjuvant chemotherapy preferably with FOLFIRINOX [2, 3]. While periprocedural mortality is between 6 and 9.8% at high-volume centers in Germany [4, 5], morbidity ranges from 30 to 50% [6]. The most common complication following pancreatoduodenectomy (PD) is delayed gastric emptying (DGE), occurring from 19 to 61% of cases [5, 6]. DGE is not life-threatening and often self-limiting [7], however, DGE is known to increase hospital stay [8] and to affect long-term cancer specific survival. This might be due to a failure to complete the full course of adjuvant therapy, which can be caused by weight loss and a poor nutritional status [9]. A wide range of causative mechanisms has been proposed for DGE including pylorospasm and preoperative cholangitis [8, 10] and most studies on DGE focused on the extent of resection and on the technique of reconstruction but failed to show superior strategies. Neither pylorus resection and -preservation [11], single- or double loop reconstruction [12] nor ante- or retrocolic reconstruction [13] with either infra- or supracolic reconstruction [14] influences the frequency of DGE after PD.

The data about smoking and its active component nicotine in relation to gastric motility are rare and have been inconsistent. There are studies showing accelerated gastric emptying in active smokers [15–17], however there are also studies in which active smoking delayed gastric emptying [18–21]. Retrospective analyses investigating risk factors in general for DGE after PD also show controversial results. While some only show an effect on DGE development using modified criteria [22], others do not put smokers at higher risk, however, they do not report on active smoking habits [23]. As of now the effect of smoking on gastric emptying after PD has not yet been investigated in detail.

## Methods

295 patients at our department underwent PD between January 2009 and December 2019. Patients were analyzed in relation to demographic factors, diagnosis, pre-existing conditions, intraoperative characteristics, hospital stay, mortality and postoperative complications with special emphasis on DGE.

All pancreatic resections were prospectively recorded in a pancreatic resection database with the approval of the institutional ethics committee (ethic committee of the Rheinische Friedrich-Wilhelms University Bonn, 347/13) and after obtaining written informed consent from the participants. Morbidity and mortality were documented according to the Dindo–Clavien-classification [24]. PF, PPH and DGE were classified according to the definitions of the International Study Group on Pancreatic Surgery [6, 25, 26].

Perioperative management was carried out according to our institutional standard operating procedure protocol. Preoperative every patient with a potential malignant tumor was discussed in our multidisciplinary Tumor Board. If patients showed signs of malnutrition, sip feeds were provided at least one week prior to surgery, parenteral nutrition was only administered when the oral route was inaccessible. Patients did not receive bowel preparations and were permitted liquids up to 2 h and solid food up to 6 h before surgery. Postoperative analgesia was provided by a mid-thoracic peridural catheter, in case of contraindications or catheter dysfunction, a patient-controlled analgesia with opioids was considered an alternative. Anesthesia was carried out according to guidelines (postoperative nausea and vomiting prophylaxis if required, near zero fluid balance, transfusion according to patient blood management guidelines and close glycemic control).

PD was performed by four certified senior pancreatic surgeons (JCK, SM, TRG, NS). After bilateral subcostal incision and complete abdominal exploration with exclusion of distant metastases or irresectable tumor, resection was carried out in a standardized fashion. Duodenoenterostomy, pancreatogastrostomy and end-to-end choledochojejunostomy were carried out as previously described [27, 28]. In brief, reconstruction was carried out using a single loop technique and pancreatogastrostomy by default. Duodenoenterostomy was performed by joining the duodenal remnant to the jejunum either in front (antecolic) or behind (retrocolic) the transverse colon. If the latter was chosen, transmesocolic reconstruction was carried out with the anastomosis either above (supracolic) or below (infracolic) the mesenterium of the transverse colon [14]. Only in case of direct infiltration of the antrum, classic Whipple procedure with double-loop reconstruction was carried out.

In all patients, perioperative a 14 French nasogastric tube (NGT) was placed which was subsequently removed if daily secretions were less than 500 ml. Two soft drains were placed at the sites of pancreatogastrostomy and choledochojejunostomy before closure of the abdomen.

Postoperative our patients stayed at least one night at the Intensive Care Unit. Patients were allowed to drink water on the day of surgery. Transition to normal diet followed the institutional enhanced recovery after surgery (ERAS)-protocol: liquid food on POD2, POD3 fat reduced/easily digestible, POD4 fiber reduced/easily digestible, POD5 basic diet (no pulses/no brassica), POD6 normal diet. In case of normal amylase levels in drainage fluid drains were removed between POD3 and 5. Only, if PF was detected octreotide (100 μg 3×/d s.c.) was administered. If bowel movement had not yet occurred by POD2, oral laxative (magnesium sulfate) was administered. In case of vomiting a NGT was re-inserted. A perioperative antibiotic prophylaxis with an aminopenicillin plus β-lactamase inhibitor and a weight adapted thrombosis prophylaxis were given to all patients.

Data were recorded and analyzed with Excel 2013 (Microsoft Corporation, Redmond, Washington, USA) and SPSS 24 (IBM Corporation, Armonk, New York, USA). Statistical analyses were carried out as described in one of our previous studies [28]: continuously and normally distributed variables were expressed as medians ± standard deviation and analyzed using student’s t test, while non-normally distributed data were expressed as medians and interquartile range and analyzed using the Mann–Whitney U test. Categorical data were expressed as proportions and compared with the Pearson x^2^ or the Fisher’s exact test as appropriate. Factors with P < 0.1 in the univariate analysis were included in multivariate stepwise logistic regression analysis. The relative risk was described by the estimated odds ratio with 95% confidence intervals. A P-value < 0.05 was considered statistically significant.

## Results

274 patients were included in the study and analyzed regarding their smoking habits (non or former smokers, n = 88, 32.1% vs. active smokers, n = 186, 68.6%). Excluded were patients for whom no information about their smoking habits was available (n = 3), patients who had had gastric resection before (n = 4) and patients with prolonged postoperative resumption to normal diet independently from DGE (long-term ventilation > 7 days, fasting due to pancreatic fistula) (n = 14). Thus, in total, 274 patients were included in our study and analyzed regarding to their smoking habits (non or former smokers, n = 88, 32.1% vs. active smokers, n = 186, 68.6%).

Smokers were younger than non-smokers (61 years vs. 69 years, p ≤ 0.001) and mainly male (73% male vs. 27% female). Preoperative conditions such as body mass index (BMI), weight loss, existence of diabetes mellitus (pre- or postoperatively), preoperative biliary drainage and cholangitis and further comorbidities included and measured by the Charlson Morbidity Index did not differ between both groups (Table 1). Active smoking patients showed significantly more pre-existing pulmonary conditions (19% vs. 8%, p = 0.002) and a significantly higher frequency of alcohol abuse was observed amongst them (48% vs. 23%, p ≤ 0.001). 58 out of 88 smokers (66%) and 147 out of 186 of non-smokers (79%) showed malign tumors (p = 0.019).

**Table 1:** Demographic and perioperative data

	Smokers	Non-smokers	P
	n = 88	n = 186	
Age (a)	61 (51.3–67)	69 (62–75)	≤ 0.001
Gender female	24 (27%)	85 (46%)	0.004
BMI	25.3 (22–27.8)	24.9 (22.7–2.7)	0.569
Diagnosis			
Malignant	58 (66%)	147 (79%)	0.019
Weight loss	54 (61%)	95 (51%)	0.131
Alcohol abuse	42 (48%)	42 (23%)	≤ 0.001
Diabetes mellitus			
Preoperative	27 (31%)	51 (27%)	0.576
Postoperative	38 (43%)	66 (35%)	0.220
Pulmonary preexisting conditions	17 (19%)	15 (8%)	0.002
Preoperative biliary drainage	43 (49%)	98 (53%)	0.554
Cholangitis	19 (22%)	41 (22%)	0.933
Charlson comorbidity index	2 (0–3)	2 (1–3)	0.394
Duration of operation (min)	431 (330.5–495)	393.5 (333.8–471.8)	0.337
Transfusions (red blood cells)	0 (0–2)	0 (0–2)	0.501
Blood loss (ml)	800 (500–1237.5)	600 (400–1000)	0.039
Venous resection	10 (11%)	33 (18%)	0.170
Multivisceral resection	6 (7%)	12 (6%)	0.909
Single loop reconstruction	61 (69%)	135 (73%)	0.486
Infracolic reconstruction	14 (16%)	45 (24%)	0.145
Retrocolic duodenoenterostomy	75 (85%)	164 (87%)	0.356
Pylorus-preserving procedure	60 (68%)	139 (74%)	0.256
Stay in hospital (d)	20 (14–28)	21 (15.8–28)	0.174
Stay in intensive care unit (d)	2 (1–4)	2 (1–4)	0.677
Stay in intensive care unit with respirator (d)	0 (0–1)	0 (0–1)	0.586

Data are shown as frequency (%) or median (interquartile range), BMI = body mass index, alcohol abuse was defined as a harmful consumption of more than 24 g of alcohol/day in male and more than 12 g of alcohol/day in females, respectively, according to the German Drug Abuse Center [29]

Intraoperative data such as the duration of the operating procedure or the need for venous or multivisceral resections were equal in both groups (Table 1). Particularly, we did not observe a difference between single- or double loop, infra- or supracolic, retro- or antecolic reconstructions nor between pylorus-preserving and classical Whipple procedures between both groups. We did observe more blood loss in smokers (800 [500–1237.5] vs. 600 [400–1000], p = 0.039), however administration of erythrocyte concentrates did not differ between both groups (0 [0–2] vs. 0 [0–2], p = 0.501). Postoperatively, the groups were comparable regarding the duration of the in hospital stay as well as the stay in the intensive care unit (Table 1).

Postoperative complications were equally distributed between both groups (Table 2). Smokers and non-smokers showed comparable occurrence of PPH and PF as well as a comparable rate of insufficiencies of biliodigestive anastomosis (BDA) or duodenoenterostomy (DE). Suprafascial wound infections were observed—at least by trend—slightly more often in active smoking patients (30% vs. 19%, p = 0.06) whereas no difference regarding intraabdominal infections with intraabdominal fluid collections could be observed. Both groups were comparable regarding major postoperative complications (Clavien III–IV) and mortality. However, the occurrence of DGE was significantly reduced in smokers (40% vs. 53%, p = 0.046). Further subclassification in DGE A, B, C or B/C did not show any difference between both groups (Table 2).

**Table 2:** Postoperative outcome/complications

	Smokers	Non-smokers	P
	n = 88	n = 186	
PPH grade B/C	17 (19%)	53 (28%)	0.104
PF grade B/C	17 (19%)	30 (16%)	0.513
Insufficiency of BDA	3 (3%)	11 (6%)	0.288
Insufficiency of DE	3 (3%)	6 (3%)	0.595
Wound infection (suprafascial)	26 (30%)	36 (19%)	0.06
Intraabdominal fluid collection	14 (11%)	20 (11%)	0.227
Clavien major (grade III–IV)	38 (43%)	89 (48%)	0.469
Mortality	4 (5%)	10 (5%)	0.514
Delayed gastric emptying	35 (40%)	98 (53%)	0.046
Grade A	18 (20%)	54 (29%)	0.132
Grade B	7 (8%)	30 (16%)	0.65
Grade C	15 (8%)	10 (11%)	0.376
Grade B/C	17 (19%)	44 (24%)	0.42

Data are shown as frequency (%), *PPH*  postpancreatectomy hemorrhage, *PF*  pancreatic fistula, *BDA*  biliodigestive anastomosis, *DE*  duodenoenterostomy

Smokers were able to tolerate solid food significantly quicker than non-smokers (POD7 vs. POD10, p = 0.004) (Table 3). The duration of intraoperative administered NGT, the last day of NGT or the rate of re-insertion of a NGT were comparable between smokers and non-smokers. Also, the duration of the application of parenteral nutrition was equally distributed in both groups.

**Table 3:** Delayed gastric emptying—parameters according to ISGPS

	Smokers	Non-smokers	P
	n = 88	n = 186	
First day of solid food intake	7 (6–11.75)	10 (7–14)	0.004
Intraoperative gastric tube (d)	3 (2–6)	4 (2–6.75)	0.444
Reinsertion of gastric tube	18 (20%)	50 (27%)	0.211
Last day of gastric tube (d)	5.5 (3–15)	10 (4–19.5)	0.176
Parenteral nutrition (d)	3 (0–5)	3 (0–8)	0.467

Data are shown as frequency (%) or median (interquartile range)

In univariate analysis, the following factors qualified for multivariate analysis: active smoking, pulmonary preexisting conditions and sex (Table 4). Active smokers were less at risk to develop DGE (p = 0.051) whereas patients with pulmonary preexisting conditions were at higher risk for developing DGE (p = 0.011).

**Table 4:** Risk factors for the development of delayed gastric emptying

	Odds ratio	95%-CI	P
*Univariate*			
Active smoking	0.593	0.354–0.992	0.046
Pulmonary preexisting conditions	2.456	1.101–5.477	0.025
Gender (male)	0.648	0.398–1.054	0.080
*Multivariate*			
Active smoking	0.539	0.290–1.003	0.051
Pulmonary preexisting conditions	2.962	1.282–6.842	0.011

*CI* confidence interval

## Discussion

To our knowledge this is the first study investigating the effect of smoking on DGE after PD. Studies that concentrated on the mere effect of smoking and nicotine on gastric motility so far have been inconclusive [15–21]. These studies were conducted 20 to 30 years ago and mainly used radioactive tracing methods to validate gastric motility. They were designed in a prospective approach, however Patient numbers included in these studies were quite low ranging from 7 to 24 and only healthy volunteers with an intact gastrointestinal tract were included. More recent retrospective cohort studies that involved patients having received PD and were concentrating on identifying general risk factors for DGE however also remain controversial [22, 23, 30, 31] since some of them identified smokers to be at higher risk for DGE whereas others did not detect an influence of smoking on developing DGE. Eisenberg et al. and Robinson et al. identified patients with a smoking history to be at higher risk for DGE [22, 23]. However, in both studies smoking history was defined as having ever used tobacco products, however they did not distinguish between active and non-smoking patients in their multivariate analysis. In another retrospective cohort study comprising over 10,000 patients, non-smoking patients were associated with DGE on a bivariate analysis compared to active smoking patients and on multivariate analysis active smokers were less likely to develop DGE. In this study patients with intraabdominal abscesses or PF were excluded in order to achieve information about primary DGE [31]. This is in line with our data. We observed significantly less DGE in active smoking patients, at the same time factors known to influence gastric motility and thus leading to secondary DGE such as diabetes, pancreatic fistula or intraabdominal abscesses did not differ in both of our groups.

As expected smokers had significantly more pulmonary pre-existing conditions than non-smokers and we observed less alcohol abuse in active smoking patients. This came rather as a surprise to us since it is established knowledge that active smokers consume more alcohol than non-smokers and active smokers are at greater risk to become alcohol dependent [32]. Amongst active smokers less malignant tumors were diagnosed than amongst former or non-smokers. This observation seemed quite interesting to us since smoking is a major risk factor for PDAC [33] and malignant diagnosis was considered to be a risk factor for developing DGE—at least after distal pancreatectomy in one of our previous studies [34]. Also, we observed more intraoperative blood loss in smokers than in non-smokers, however the amount of intraoperative blood loss does not influence the occurrence of DGE [8, 22, 23] and we did not observe any difference in the rate of blood transfusion which is known to be associated with prolonged hospital stay and increased mortality [30, 35].

One of the main and exciting findings of our study is the fact that active smoking patients tolerated solid food intake more quickly than non- or former smokers. The first day of returning to a normal diet is considered a valid parameter in defining DGE [6], parameters that are related to NGT such as the duration of intraoperative administered NGT, the last day of NGT or the rate of re-insertion of a NGT did not differ in our study between smokers and non-smokers. These parameters have to be interpreted more carefully since removing of NGT often is not exclusively determined by the extent of DGE but also depends on other factor such as removal of NGT by accident or patient request. Especially in a retrospective study design, as in our study, we consider these parameters as quite helpful for specifying DGE but would not solemnly rely on them.

It is well known that DGE prolongs hospital stay [8]. In this study active smokers were less at risk to develop DGE however in our cohort, this did not lead to a reduction in the overall length of the hospital stay. In another retrospective study analyzing factors that were associated with a short length of stay after PD, more smokers were discharged before or on POD5 [36]. However, in this study, smokers were defined as current or former smokers whereas the present study distinguishes between active smoking patients and former- or non-smoking patients and thus patient cohorts are not comparable. Factors that were associated with an extended hospital stay after PD such as BMI < 25 kg/m^2^, type of surgical procedure, blood transfusion over 3 U and fluid input > 57 ml/kg [30] with the exception of age over 60 years, did not differ between both groups in our study. Regarding the effect of minimally invasive PD on DGE development, the findings are inconclusive. National registries [37] and meta-analyses [38, 39] were not able to detect an effect, single-center studies showed different results (40, 41). Robotic PD was introduced in our department several years ago, but because its use does not span the whole period, we did not include minimally invasive PD into our analysis. Knowing the shortcomings of retrospective and single-center design, further observations as randomized, controlled multicenter studies including robotic PDs need to follow.

## Conclusions

DGE remains the most common complication after PD. Our data show that DGE occurs less common in active smokers and they tolerate solid food intake more quickly than non-smokers. Further observation studies and randomized, controlled multicentre studies without the deleterious effect of smoking, for instance by administration of a nicotine patch, are needed to examine if this effect is due to nicotine administration.

## Data Availability

Our anonymized pancreatic resection database contains sensible data (e.g. date of surgery), with which certain patients could be identified. According to German law and according to the approval of the ethics committee, these data must not be published. Access to the database can be obtained from the corresponding author upon reasonable request.

## Abbreviations

BDA: Biliodigestive anastomosis
BMI: Body mass index
DE: Duodenoenterostomy
DGE: Delayed gastric emptying
NGT: Nasogastric tube
PD: Pancreatoduodenectomy
PDAC: Pancreatic ductal adenocarcinoma
PF: Pancreatic fistula
PPH: Post pancreatectomy haemorrhage
POD: Postoperative day
